# Methodological Considerations Regarding Diuretic Intensity Score and Mortality in Hospitalized Heart Failure Patients

**DOI:** 10.1002/clc.70403

**Published:** 2026-06-29

**Authors:** Noor‐ul‐Eman Haider, Uzair Ali Khan, Laraib Saleem, Masood Khan, Muhammad Hassan Fayaz

**Affiliations:** ^1^ Khyber Medical College Peshawar Khyber Pakhtunkhwa Pakistan; ^2^ Doctors PolyClinic Kasur Punjab Pakistan

Dear Esteemed Editor,

The investigation by Zhou et al., focused on exploring the relationship between Diuretic Intensity Score (DIS) and the probability of short‐term survival increase among heart failure patients, is particularly interesting for us due to the new approach introduced by the scientists regarding the assessment of the treatment intensity [1]. Despite the fact that the development of the standardized measures of the exposure level to the drug under analysis can be regarded as an innovation in the field, there are two possible methodological problems that might have an adverse impact on the accuracy of the results provided by the scientists.

The first problem concerns the possibility of the existence of exposure definition bias that can be compared to immortal time bias [2]. The distinction between the traditional exposure definition and the DIS score estimation is the fact that the latter depends on the amount of exposure to the medicine during the patient's stay at the hospital. It means that only those patients who were placed into the moderate and severe categories were alive long enough to escalate their therapy and use more diuretics or intravenous therapy [3]. It is especially critical taking into account the large scale of estimated hazard ratios. Thus, the hazard ratio for the most severe DIS group turned out to be exceptionally large, which would result in a more pronounced decrease in mortality compared with other modern approaches to treating heart failure. The reason why it can be explained by the so‐called “survival paradox in an exposure‐defined setting” since the real impact is due to various probabilities of treatment but not to its effectiveness [4, 5]. Despite the possible survivorship bias mentioned by the authors themselves, the inability to perform a time‐varying exposure analysis makes its detection difficult [6].

The second point to consider in the context of the current research is the issue of DIS construct validity. Prognostic markers should provide maximum predictive accuracy in the clinical environment, and the capability of such markers to identify biological differences among patients in terms of their response to the treatment and additional prediction value as compared to other markers should be evaluated [7]. When it comes to DIS, one has to mention various dimensions associated with the therapy including the mode of drug delivery, drugs employed, intensity of the treatment, and combination of treatment. All those dimensions are empirically weighted, but there is no explanation as for why those weights were selected in the framework of pharmacological approach. The main thing is that the predictive power in the current study does not indicate superiority of empirically weighted DIS to simply the number of prescribed diuretics. Thus, the question arises whether this weighting involves some knowledge about the disease or is a proxy for complexity of the treatment. DIS may include not only information on the intensity of treatment but also physician preference, and so on [8].

Finally, although the DIS is a breakthrough program for standardizing the effect of diuretic treatment, the possibility of bias in defining the exposure as well as the problem related to construct validity may account for most of the relation that has been identified between mortality and diuretics. Further studies using time‐dependent analysis will shed light on this matter.

## Author Contributions

All authors contributed equally to this work.

## Funding

The authors have nothing to report.

## Ethics Statement

The authors have nothing to report.

## Consent

The authors have nothing to report.

## Conflicts of Interest

The authors declare no conflicts of interest.

## Data Availability

Data sharing not applicable to this article as no datasets were generated or analyzed during the current study.
